# Molecular identification in metabolomics using infrared ion spectroscopy

**DOI:** 10.1038/s41598-017-03387-4

**Published:** 2017-06-13

**Authors:** Jonathan Martens, Giel Berden, Rianne E. van Outersterp, Leo A. J. Kluijtmans, Udo F. Engelke, Clara D. M. van Karnebeek, Ron A. Wevers, Jos Oomens

**Affiliations:** 1 Radboud University, Institute for Molecules and Materials, FELIX Laboratory, Toernooiveld 7c, 6525ED Nijmegen, The Netherlands; 2 Department of Laboratory Medicine, Translational Metabolic Laboratory, Radboud University Medical Center, Nijmegen, The Netherlands; 3 Department of Pediatrics, BC Children’s Hospital Research Institute, Centre for Molecular Medicine and Therapeutics, University of British Columbia, Vancouver, British Columbia Canada; 4 van’t Hoff Institute for Molecular Sciences, University of Amsterdam, 1098XH Amsterdam, Science Park 908, The Netherlands

## Abstract

Small molecule identification is a continually expanding field of research and represents the core challenge in various areas of (bio)analytical science, including metabolomics. Here, we unequivocally differentiate enantiomeric N-acetylhexosamines in body fluids using infrared ion spectroscopy, providing orthogonal identification of molecular structure unavailable by standard liquid chromatography/high-resolution tandem mass spectrometry. These results illustrate the potential of infrared ion spectroscopy for the identification of small molecules from complex mixtures.

## Introduction

The characterization of metabolites in a biological system, metabolomics, is used for biomarker identification related to many diseases, including cancer^1^, Alzheimer’s disease^2^, inborn errors of metabolism^3, 4^, and others. Increasingly, untargeted high-resolution mass spectrometry (MS) plays a leading role in the identification of small-molecule metabolites, such as organic (amino) acids, saccharides, and other biomarkers. However, in many cases definitive identification of molecular structures is difficult using standard MS-based methods alone and orthogonal techniques are required. This can be exemplified by focusing on saccharides, which play an important role in various biochemical processes including the regulation of biochemical pathways and cellular interaction. Saccharides exhibit uniquely complex structural diversity, largely due to their enantiomeric building blocks which are isobaric (identical m/z) and thus challenging to characterize by mass spectrometry^5, 6^. These building blocks (monosaccharides), such as hexoses, N-acetylhexosamines and deoxyhexoses can be distinguished from each other based on their unique m/z values, but the actual identity of the hexose or N-acetylhexosamine monosaccharide itself is difficult to obtain and must often be inferred by assumptions based on glycan biosynthetic principles^7^.

Recently, our group discovered N-acetylmannosamine (ManNAc) as a biomarker for NANS-deficiency^8^, a new inborn error of metabolism in sialic acid metabolism. However, the differentiation of N-acetylmannosamine from other possible enantiomeric N-acetylhexosamines found at the same m/z, is impossible using standard operating HPLC-MS/MS protocols in most bioanalytical laboratories (including our own), as they cannot be separated using reversed-phase chromatography and have identical MS/MS fragmentation spectra. Indeed, for the definitive identification of ManNAc from patient body fluids, it was necessary to use NMR techniques requiring significantly larger sample volumes and having significantly lower sensitivity than MS-based methods^8^. Previous studies have demonstrated the ability to distinguish monosaccharides using MS alone, but have mainly relied on either following complex MS^>2^ dissociation channels, often based only on differences in the relative peak intensities of common fragmentation channels^9–11^, or derivatization schemes that are difficult to apply to complex biological samples^12, 13^.

Infrared ion spectroscopy (IR-IS) has seen widespread use over the past decade for the identification of (bio)molecular structures in the setting of academic physical chemistry labs^14–22^. Using a tuneable infrared laser such as the FELIX IR free electron laser^19, 23^, an IR spectrum can be generated for any m/z peak isolated from the mass spectrum. Here, we demonstrate the use of IR-IS to distinguish three N-acetylhexosamines directly from urine and cerebrospinal fluid (CSF) without any sample preparation (aside from dilution) or chromatographic separation. This is the first demonstration of the use of IR-IS for the identification of small molecules from complex mixtures. These results demonstrate the potential and natural extension of IR-IS to applied (bio)analytical chemistry.

**Figure 1:**
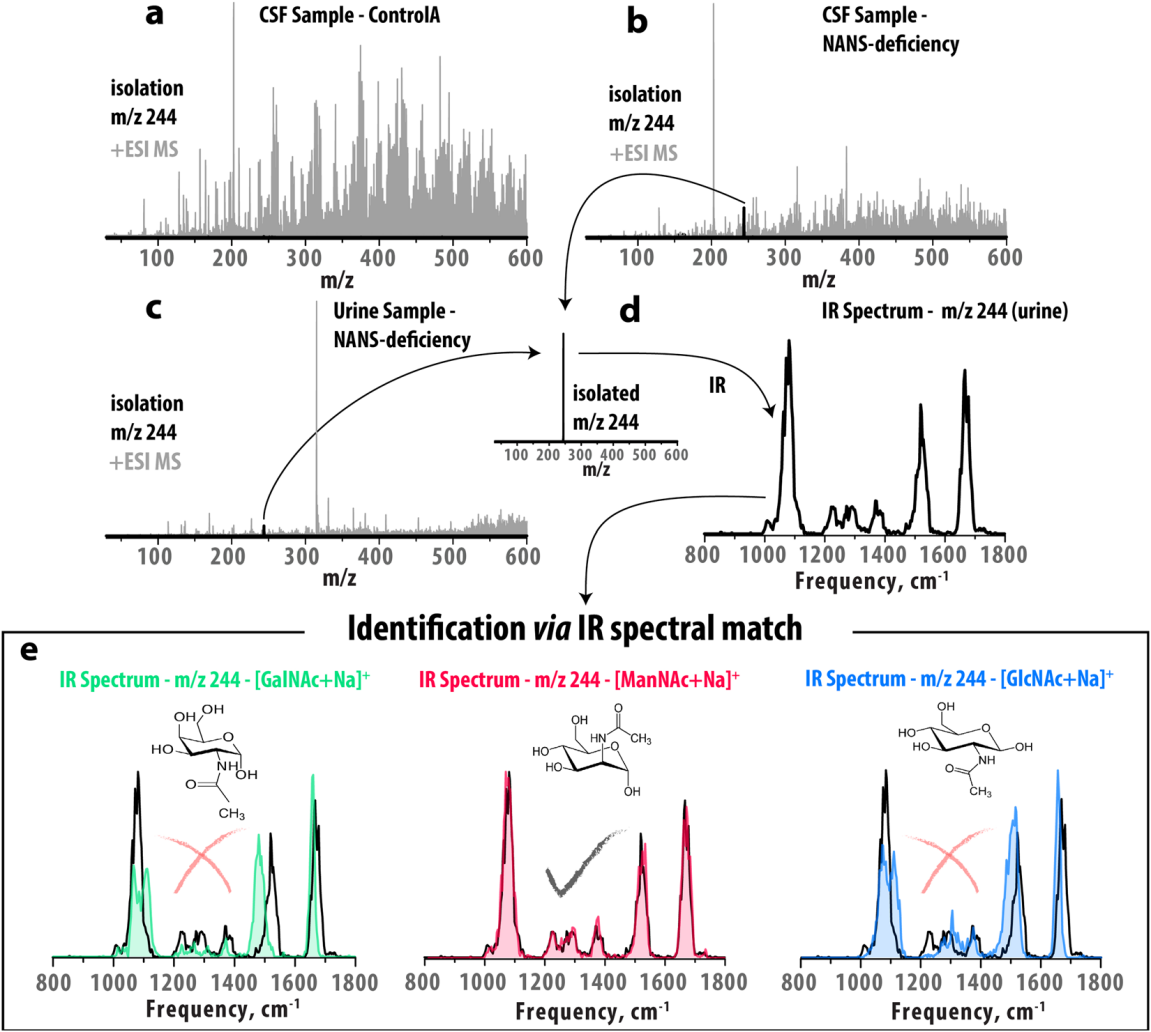
Experimental approach of IR-IS, combining mass spectrometry with infrared spectroscopy for metabolite identification. In panels (**a**–**c**), grey spectra are full +ESI MS spectra of body fluid samples and black spectra are generated after isolation of ions at m/z 244. Panel (**a**) shows the MS spectrum of a control CSF sample demonstrating the absence of ions corresponding to the m/z value expected for ManNAc ([M + Na]^+^ m/z 244), the targeted biomarker in these experiments. Panels (**b**) and (**c**) contain MS spectra generated from (**b**) a CSF sample and (**c**) a urine sample from a patient having NANS deficiency. Panel (**d**) illustrates the mass isolation of ions at m/z 244, and the subsequent measurement of their IR spectrum in the ion trap (black trace). In panel (**e**), the route to identification is achieved via IR spectral match between the spectrum measured from the patient sample (black in all graphs) and reference spectra generated for the known model compounds of alternative N-acetylhexosamines. The IR spectra serve as structural signatures, in this case providing clear identification of the species at m/z 244 from the patient sample as ManNAc by spectral match with the reference IR spectrum (red).

IR-IS and simple direct infusion electrospray ionization (ESI) is used in a modified quadrupole ion trap mass spectrometer to demonstrate the identification of ManNAc in the patient’s body fluid samples and its distinction from the enantiomeric N-acetylhexosamines shown in Fig. 1. This approach is largely enabled by recent advances in experimental sensitivity and efficiency that allow us to measure IR spectra for ions at concentrations down to the low nanomolar range, consuming less than <50 μL of solution per IR spectrum^23^. Although the N-acetylhexosamines discussed here cannot be separated by the standard HPLC protocols in our laboratory, this sensitivity and efficiency generally allows us to measure an IR spectrum of ions generated from a collected HPLC fraction.

## Results and Discussion

In Fig. 1, we observe the level of m/z 244, corresponding to the sodium adduct [M + Na]^+^ of an N-acetylhexosamine, in the patient body fluids to be elevated in comparison to a control CSF sample (Fig. 1 panels (**a**) CSF-control *vs*. (**b**) CSF-patient and (**c**) urine-patient). Ions at m/z 244 are mass isolated and subsequently irradiated *in situ* with the tuneable infrared radiation of the IR-FEL to generate their infrared spectrum (black), shown in panel (**d**). Photodissociation occurs when the laser wavelength is resonant with one of the vibrational frequencies of the trapped ions, generating a fragment peak(s) in the mass spectrum indicative of IR absorption. The fragmentation yield is monitored while the frequency of the laser is scanned^24^. In the final step, identification of the ions at m/z 244 from the body fluid sample is achieved by comparison with reference IR spectra obtained through analogous measurements for standard solutions of known reference compounds.

Panel (**e**) of Fig. 1 presents these comparisons where the IR spectrum of the ions at m/z 244 measured from the patient body fluid (black all graphs) can clearly be assigned to N-acetylmannosamine (middle, red) and not to two other reference IR spectra belonging to alternative N-acetylhexosamine model compounds, N-acetylgalactosamine (green trace) and N-acetylglucosamine (blue trace). Figure S1 demonstrates that GlcNAc and GalNAc are also distinguishable from each other on the basis of their IR spectra. Since the presumably high levels of sodium in the body fluids result in observation of exclusively the sodium adduct of ManNAc, we generated the sodium adduct for the model compounds as well by addition of 10^−6^ M Na^+^ to the reference solutions.

In addition to identification based on matching to reference measurements from model compounds, basic functional group information is also easily inferred from the IR spectra of unidentified ions in a mass spectrum. For example, the two features at approximately 1500 and 1675 cm^−1^ correspond to C-N and C = O stretch vibrations, respectively indicating the presence of these functional groups in the molecular structure of the mass-isolated ions. For the case of an unknown metabolite where reference compounds are not available, this gives information related to chemical structure which could help narrow down a list of database matches for isobaric species, for example unmodified or phosphorylated saccharides do not feature IR bands above 1500 cm^−1^ (except H-stretches normally found above 2800 cm^−1^). Comparison against predicted IR spectra from quantum-chemical calculations have the potential to further enhance the structural information extracted and hence further help narrow down possible database matches.

**Figure 2:**
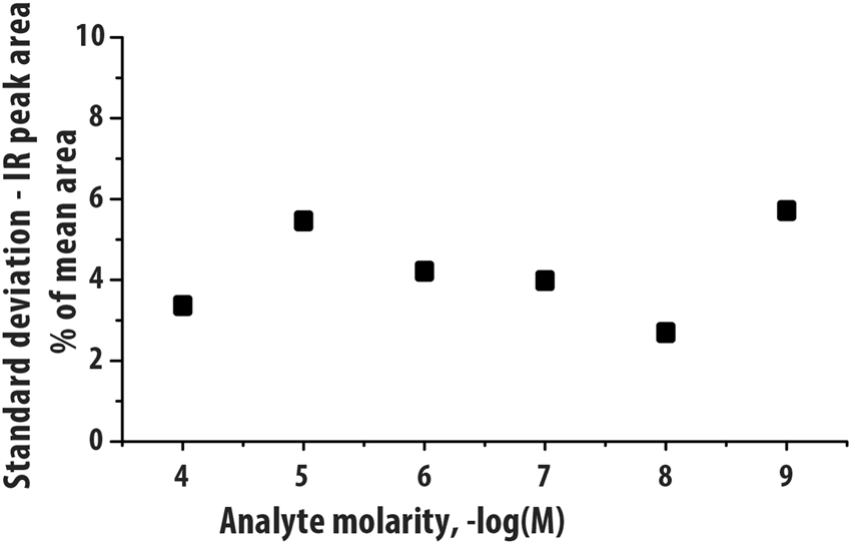
Standard deviation of the IR peak area as a percent of the mean value. Five repeated measurements over the 3500–3590 cm^−1^ range for the protonated amino acid tryptophan, a well understood system studied previously^23^, are presented. The standard deviation of IR peak area over the range of 100 μM – 1 nM has a maximum of 5.5%.

The route to identification demonstrated in Fig. 1 is based on clear but modest differences in the IR spectra of the different reference compounds. Figures S2–S4 demonstrate the high degree of reproducibility of IR-IS measurements, over repeated scans, from different sample matrices (urine and CSF) and with varying concentration, providing clear validation of the spectral match in Fig. 1. In order to quantify the reproducibility of these measurements, Fig. 2 presents a comparison of the standard deviation in peak areas of IR yields measured for a well-known model system^23^, the protonated amino acid tryptophan (m/z 205). Five identically repeated IR-IS measurements (in this case using a table-top optical parametric oscillator (OPO) mid-IR laser system in the 3500–3590 cm^−1^ spectral region) were made at each of six concentrations between 1 nM and 100 μM. We compare the standard deviation of the IR peak areas between the five measurements (as a percentage of the mean peak area) and find less than 5.5% deviation over the full concentration range. Note that by considering the fragmentation yield as the IR intensity (see methods section), we compare the fraction of ions that dissociate at each frequency point, making the measurements independent of the total ion count and hence of concentration.

While the IR-FEL is unique in its wide tuneability and high output power, table-top mid-IR laser systems (especially OPOs) can also be used (especially in the ~2800–3800 cm^−1^ spectral region where they probe hydrogen stretching vibrations) for IR-IS experiments^18, 23, 25^. However, the output power of OPOs is substantially lower than that of IR-FELs, somewhat limiting their applicability to ions having a relatively low dissociation threshold. Figure S5 presents the distinction of the three N-acetylhexosamines discussed above using an OPO mid-IR laser system, demonstrating that they are as well each differentiated by their H-stretching vibrations in the 3200–3800 cm^−1^ region.

The results presented above demonstrate the (bio)analytical potential of IR-IS for the identification of small molecules from complex matrices in analytical mass spectrometry, offering a new orthogonal characterization technique. Additionally, we envision the workflow demonstrated here to be applicable in a variety of fields, including plant metabolomics, pharmacology, environmental sciences, and others. In medicine, early diagnosis enabling treatments of genetic conditions such as NANS deficiency is essential. Thus with advances in therapies, it is clear that IR-IS will play an important role for the identification of conditions with challenging biomarkers.

## Methods

### Infrared Ion spectroscopy

**Figure 3:**
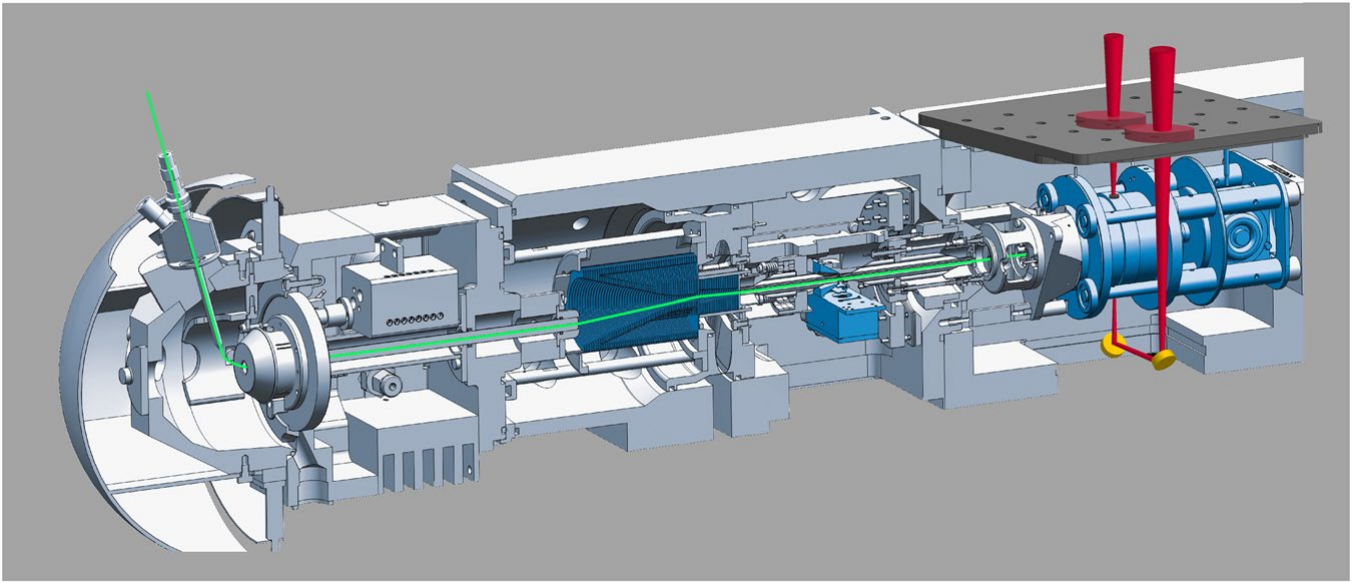
An Ion trap mass spectrometer modified for infrared ion spectroscopy. Gas phase ions are generated by direct infusion electrospray ionization and are transferred (green) to the ion trap where ions of a single m/z value are isolated and stored. Subsequently, a tunable infrared laser (red) irradiates the ions and generates a frequency-dependent photodissociation spectrum as the laser frequency is tuned.

IR-IS measurements were performed using a modified quadrupole ion trap mass spectrometer (Bruker, amaZon Speed ETD) that has been coupled to the IR beam line of the FELIX free electron laser, illustrated in Fig. 3 ^19, 23^. The FELIX laboratory (http://www.ru.nl/felix/) operates as an international user facility where beam time is awarded through biannual calls for proposals open to external researchers. Hardware modifications to the mass spectrometer to give optical access to the trapped ions include the introduction of a new ring electrode with 3 mm holes at its top and bottom, the introduction of mirrors below the trap to guide the IR-laser beam back out of the vacuum housing (onto a pyroelectic sensor measuring the pulse energy) and IR-transparent optical windows in the vacuum chamber. Details of these modifications and of software operation have been previously reported elsewhere^23^. Body fluid samples were diluted to give ManNAc concentration at ~10^−6^ M in order to sufficiently dilute the higher concentration components in the sample matrix, while maintaining a favourable signal to noise ratio. The diluted samples were directly electrosprayed (+ESI) to produce IR/mass spectra. All [M + Na]^+^ ions for reference measurements were generated by +ESI from solutions of 10^−6^–10^−8^ M (in 50:50 acetonitrile:H_2_O) containing NaCl at 10^−6^ M. Reference compounds and electrospray solvents were obtained from Sigma-Aldrich.

For these experiments, the FELIX free electron laser provided infrared radiation in the 800–1800 cm^−1^ range as 5 μs macropulses (at a 10 Hz repetition rate) of 30–60 mJ per pulse (the bandwidth is ~0.4% of the center frequency). A table top infrared optical parametric oscillator (OPO) provides infrared radiation in the 3200–3800 cm^−1^, producing 10–15 mJ per 5 ns pulse with a bandwidth of 3 cm^−1^ (LaserVision, USA). When the IR photons are resonant with a vibration in the trapped ions the absorption occurs leading to an increase in the internal energy of the ion. Aided by intramolecular vibrational redistribution of the absorbed energy, the ions dissociate once they reach the limit of the dissociation channel with the lowest threshold. Typically, after a single macropulse, dissociation occurs and generates a frequency-dependent fragment ion signal in the mass spectrum. By relating the parent and fragment ion intensities (yield = ΣI(fragment ions)/ΣI(all ions)) as a function of the IR frequency, an infrared vibrational spectrum is generated. The yield at each IR point is obtained from typically four to eight averaged mass spectra and is linearly corrected for laser power; the frequency is calibrated online throughout the measurements using a grating spectrometer (IR-FEL) or a wavemeter (OPO).

## Electronic supplementary material

Supplementary Information
