# Supplementary material for: Molecular identification in metabolomics using infrared ion spectroscopy
Source: Sci Rep. 2017 Jun 13;7:3363. doi: 10.1038/s41598-017-03387-4 (PMC5469762; doi:10.1038/s41598-017-03387-4)
Supplement: Supplementary file 1 — Supplementary Information [file 41598_2017_3387_MOESM1_ESM.pdf]

Supporting information

Molecular identification in metabolomics using infrared ion spectroscopy

Jonathan Martens[1*], Giel Berden[1], Rianne E. van Outersterp[1], Leo A. J. Kluijtmans[2], Udo F. Engelke[2], Clara D. M. van Karnebeek[3], Ron A. Wevers[2*], Jos Oomens[1,4*]

[1]*Radboud University, Institute for Molecules and Materials, FELIX Laboratory, Toernooiveld 7c, 6525ED Nijmegen, The Netherlands*
[2]*Department of Laboratory Medicine, Translational Metabolic Laboratory, Radboud University Medical Center, Nijmegen, The Netherlands*
[3]*Department of Pediatrics, BC Children's Hospital Research Institute, Centre for Molecular Medicine and Therapeutics, University of British Columbia, Vancouver, British Columbia, Canada*
[4]*van't Hoff Institute for Molecular Sciences, University of Amsterdam, 1098XH Amsterdam, Science Park 908, The Netherlands*

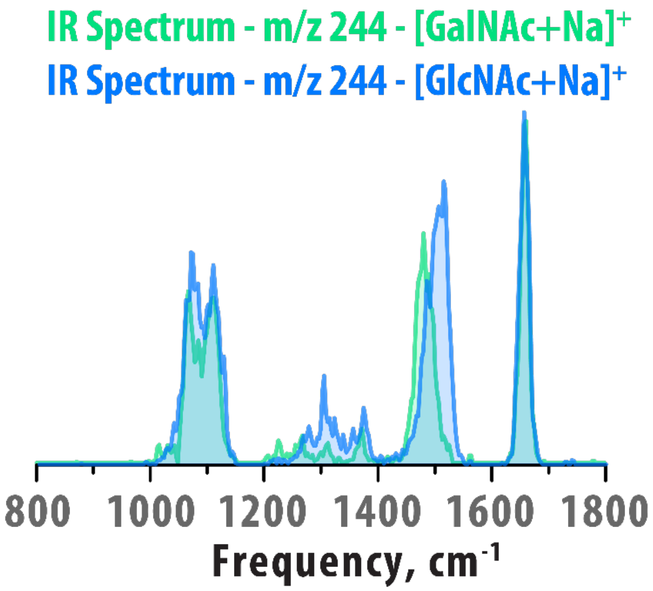

Figure S1 | IR spectra of GalNAc and GlcNAC reference model compounds illustrating that each of the three N-acetylhexosamines are distinguishable from one another on the basis of the IR spectra.

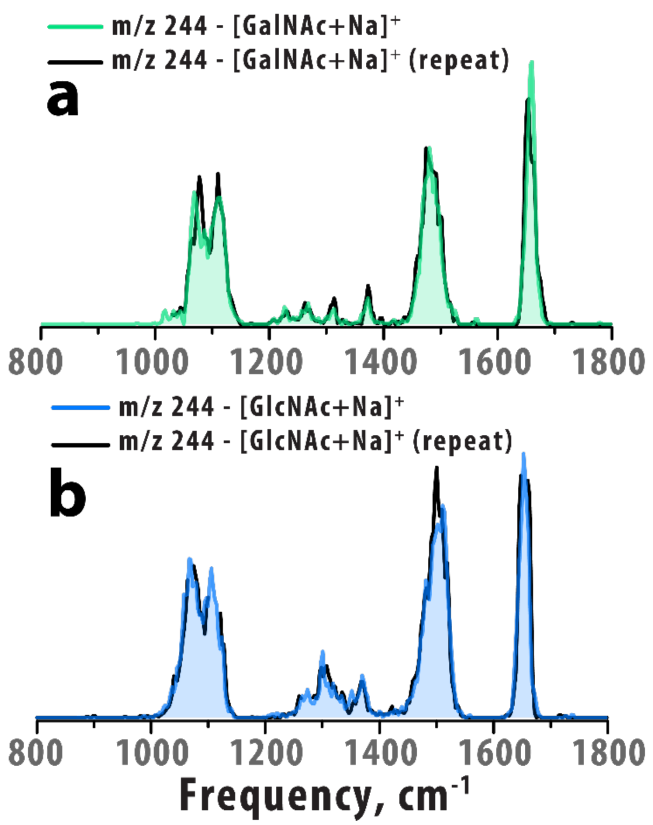

Figure S2 | Panels (a) and (b) present duplicate IR spectra measured for the GalNAc and GlcNAc reference model compounds, respectively, demonstrating the reproducibility of the distinctive features of their IR spectra in both cases.

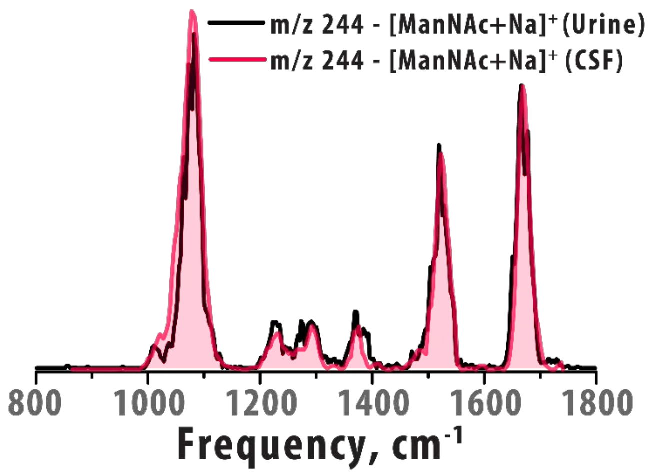

Figure S3 | The IR spectrum of ManNAc measured after direct infusion +ESI of a diluted urine sample (black) and a cerebrospinal fluid sample (CSF, red).

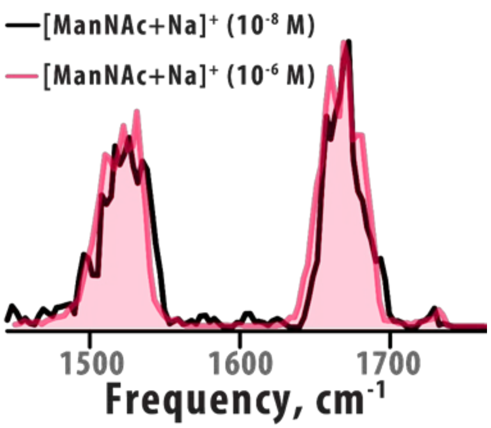

Figure S4 | Partial IR spectrum of the ManNAc reference compound measured at 10^{-8} M (black) and at 10^{-6} M (red), demonstrating that IR spectra are largely concentration independent over a wide range.

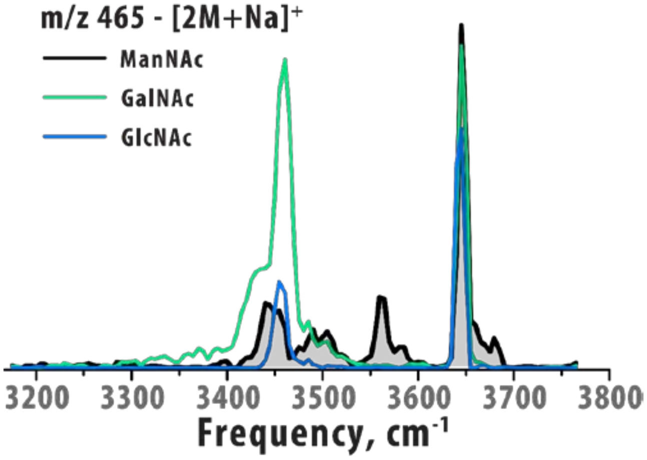

Figure S5 | IR spectra of $[2M+Na]^+$ ions from the three N-acetylhexosamines discussed in Figure 1 generated using an OPO IR source in the 3200-3800 cm^{-1} region. The $[2M+Na]^+$ dimer complex ions were selected because the $[M+Na]^+$ monomer ions did not show sufficient fragmentation after irradiation with the relatively low-power OPO output (<15 mJ pulse energy).
